# Efficacy and Safety of Echinacoside in a Rat Osteopenia Model

**DOI:** 10.1155/2013/926928

**Published:** 2013-03-20

**Authors:** Xiaolin Yang, Fei Li, Yanan Yang, Jinyang Shen, Run Zou, Panpan Zhu, Chunfeng Zhang, Zhonglin Yang, Ping Li

**Affiliations:** ^1^Guangdong Provincial Key Laboratory of Pharmacodynamic Constituents of TCM and New Drugs Research, Jinan University, Guangzhou 510632, China; ^2^State Key Laboratory of Natural Medicines, China Pharmaceutical University, Nanjing 210009, China; ^3^Institute of Materia Medica, Shanghai No.1 Biochemical & Pharmaceutical Company Ltd., Shanghai 200240, China

## Abstract

This study aimed to investigate the efficacy and safety of echinacoside (ECH) using an osteopenia rat model. Forty-eight 6-month-old female Sprague-Dawley rats were randomly divided into one sham-operated group (SHAM) and five OVX (ovariectomized) subgroups: SHAM with vehicle 0.5% carboxymethylcellulose sodium (0.5% CMC-Na) and OVX with vehicle (OVX), OVX with 17**β**-estradiol (E2), and OVX with ECH of graded doses (ECH-L, ECH-M, and ECH-H). The effects of ECH and E2 on serum biochemical parameters, bone mineral density (BMD), bone biomechanical properties, bone microarchitecture, and immunohistochemistry were examined, and safety assessments were also evaluated. The results showed that ECH treatments improved total femur BMD, bone microarchitecture, and biomechanical properties and decreased serum marker levels in comparison to OVX group. Moreover, ECH administration significantly increased osteoprotegerin (OPG) level, and decreased receptor activator of nuclear factor-**κ**B ligand (RANKL) level in serum, as well as in proximal femur. Importantly, ECH treatment ameliorated the lipid parameters without the overall incidences of adverse events of uterus and mammary gland compared to OVX and SHAM groups. This study demonstrated that administration of ECH for 12 weeks can effectively and safely prevent OVX-induced osteoporosis in rats via increasing the OPG/RANKL ratio.

## 1. Introduction

Osteoporosis is one kind of skeleton metabolic disorders characterized by reduction of bone mass and microarchitectural deterioration of bone tissue, which may result in skeletal fragility and fractures. It has already become one of the leading threats for the health of the aging population [1, 2], evidenced by an estimated prevalence of 200 million people worldwide and the annual attendant costs, for this disease, have exceeded approximately 10 billion dollars [3, 4]. 

Estrogen depletion disrupts bone homeostasis, altering differentiation and activity of osteoblasts and osteoclasts and therefore played a very important role in the initiating and developing of osteoporosis and it has been shown to be a major risk factor for the development of postmenopausal osteoporosis in women [5, 6]. Estradiol esters and conjugated estrogens have strong suppressive effects on osteoporotic activities in the bone [7]. Currently, administration of bisphosphonates and estrogen replacement therapy (ERT) are two main forms of treatment and prevention of osteoporosis and even reduce the incidence of fracture in postmenopausal osteoporosis women [8, 9]. However, bisphosphonates lead to atraumatic fracture of bone as a consequence of an adynamic state similar to that described in patients on chronic maintenance hemodialysis [10], and long-term estrogen treatments are accompanied by the undesired side effects, especially the higher incidence of coronary heart disease, invasive breast cancer, stroke, pulmonary embolism, endometrial cancer, colorectal cancer, and hip fracture [11]. Thus, new effective and safety treatment strategies of osteoporosis are highly needed.

**Figure 1:**
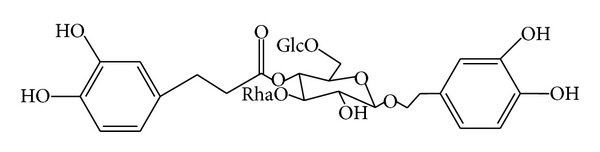
Chemical structure of echinacoside (ECH).

The *Cistanche tubulosa (Schrenk) *R. Wight (Orobanchaceae parasitic plant) is widely distributed in North Africa and Asian countries, and the stems of *C. tubulosa* are commonly used to treat kidney deficiency and neurodegenerative diseases as a promoting agent [12]. Echinacoside (ECH, Figure 1) is one of the major constituents of a famous traditional Chinese medicine, Herba *Cistanches* (the stems of *Cistanche deserticola, Cistanche salsa, *or* Cistanche tubulosa*) [13]. Many researches demonstrated that ECH, as a natural polyphenolic compound, has various kinds of pharmacological activities, such as antioxidative, anti-inflammatory, neuroprotective, hepatoprotective, nitric oxide radical-scavenging [14], and vasodilative ones [15]. Currently, there are no reports available on the therapeutic effects of ECH and its rescuing mechanisms on osteoporosis in OVX rat models.

Our previous study showed that ECH can stimulate bone regeneration through increasing OPG/RANKL ratio in MC3T3-E1 cells [16]. This suggests that ECH warrants further investigation. To further investigate this, we conducted experiments in an OVX rat model [17], a standard model for the investigation of morphological and biomechanical changes after different treatments for osteoporosis. In this report, we assessed the effects of 12 weeks of ECH treatments for biochemical parameters in serum, bone quality, bone mechanical properties, bone microarchitecture, and immunohistochemistry, as well as safety indicators including lipid parameters, carcinoembryonic antigen (CEA), and cancer antigen 125 (CA-125) levels in serum and uterus and mammary gland histology in OVX rats. Most importantly, we first reported the effect of ECH on the OPG/RANKL system through examining serum and bone OPG/RANKL levels.

## 2. Materials and Methods

### 2.1. Plant Materials and Animals

The fresh stems of the *Cistanche tubulosa (Schrenk) R. Wight*, harvested in November 2010 in Xinjiang, China, were purchased from Institute of Ecology and Geography Chinese Academy of Sciences in Xinjiang, China. The materials were identified by Professor Ping Li, School of Traditional Chinese Medicine, China Pharmaceutical University. The voucher specimen (no. 02369433) has been deposited in our laboratory in China Pharmaceutical University. All the materials were dried at the room temperature to constant weight.

Echinacoside (ECH) was separated and purified from an ethanol extract of *Cistanche tubulosa (Schenk)* R. Wight by our laboratory according to a method reported previously [18] with slight modification, and its structure was confirmed by UV, IR, MS, and NMR spectroscopy. Its purity (98.5%) was determined by Agilent 1260 Series HPLC with DAD detector (Agilent Scientific, Co., USA). 17 *β*-estradiol (E2, Sigma; purity ≥ 98%) was used as a positive control.

Forty-eight female Sprague-Dawley rats (Nantong University, China), aged 6 months with the body weight of 280 ± 20 g, were allowed to acclimatize for 7 days before the start of the experiment. Every four animals were kept in one cage with a standard laboratory diet and tap water under climate-controlled conditions (25°C, 55% humidity, and 12 h of light alternating with 12 h of darkness). All procedures were carried out in accordance with the Guide for the Humane Use and Care of Laboratory Animals and were approved by the Animals Ethics Committee of the University.

### 2.2. Pharmaceutical Treatment

Rats were randomly divided into six groups (8 rats in each group): sham operated (SHAM), bilaterally ovariectomized (OVX), OVX and E2 treatment, and three other OVX and different doses of ECH treatment groups. All rats were anesthetized via intraperitoneal (i.p.) injection of 300 mg/kg chloral hydrate (Sinopharm, China) and then ovariectomized at week 0 and the SHAM group underwent a sham ovariectomy. The surgical procedure was performed under aseptic conditions following the University of China Pharmaceutical University Animal Care protocol. Rats were left untreated for 4 weeks to allow for rats to recover and develop osteopenia. After 4 weeks, rats in the OVX and E2 group received daily intragastric (i.g.) administrations of E2 (50 *μ*g/kg/day) for 12 weeks; the sham and OVX rats were subjected to daily intragastric administration of 0.5% CMC-Na (Sinopharm, China) as vehicle. E2 and ECH were dissolved in a vehicle of 0.5% CMC-Na, and rats in the OVX and ECH groups received daily i.g. administrations of ECH (ECH-L, 30 mg/kg/day; ECH-M, 90 mg/kg/day; and ECH-H, 270 mg/kg/day) for 12 weeks, respectively. Body weight was measured weekly, and the ECH dose adjusted accordingly.

### 2.3. Animal Euthanasia and Specimen Collection

At necropsy, blood was collected from the carotid artery under general anesthesia in the early morning. The blood was allowed to clot and centrifuged at 3,000 ×g for 10 min. Serum was harvested and stored at −20°C until use for biochemical assays. After animal euthanasia, femora were isolated for BMD, Micro-CT, and biomechanical and immunohistochemical analysis, and mammary glands and uterus were removed, freed from fat, and fixed in sodium phosphate (PBS) 10% buffered formaldehyde solution (pH 7.4), and then stored at 4°C until use for histological and immunohistochemical evaluation to assess the safety of ECH.

### 2.4. Effectiveness Assessment

#### 2.4.1. Serum Biochemical Analysis

Alkaline phosphatase (ALP) is known to be associated with bone metabolism and differentiation of osteoblasts and its activity is one of the most common indicators of osteoblast differentiation and osteogenic properties [19]. Thus, the serum ALP level was determined using an ALP activity assay kit (Nanjing Jiancheng Bioengineering Institute; Nanjing, China). Tartrate-resistant acid phosphatase 5b (TRACP-5b), secreted by osteoclasts, correlates with bone resorption activity in abnormal bone metabolism [20]. For markers of bone resorption, TRACP-5b levels were measured with enzyme-linked immunosorbent assay (ELISA) kits (R&D Systems Inc.) in serum. Serum OPG and RANKL were also assayed with ELISA kits (R&D Systems Inc.). All of the measurements of ELISA kits were performed according to the protocols provided by the manufacturers.

#### 2.4.2. Bone Mineral Density Measurement

Bone mineral density (BMD) of the right total femora was measured by using Discovery W dual energy X-ray absorptiometry (DEXA, Hologic Inc., Boston MA, USA) equipped with appropriate software (edition 13.1.2) for bone density assessment in small laboratory animals. After scanning, BMD in right total femora was obtained for statistical analysis. The investigator performing the measurement was unaware of the treatments the rats had received.

#### 2.4.3. Micro-CT Bone Architecture Analysis

The representative right distal femora were scanned to evaluate three-dimensional (3D) trabecular microarchitectures using microcomputed tomography (*μ* CT-Sharp, ZKKS-MCT, China). The scanning system was set to 60 kV, 40 W, with an isotropic voxel size of 22 *μ*m. Scanned images were reconstructed using ZKKS Micro-CT 3-D analysis software of version 3.0. Specimen specific thresholds were determined by first selecting a volume of interest (VOI), generating the attenuation histogram, and determining the threshold that segments mineralized tissue from background. 100 slices (3.0 mm) of bone (secondary spongiosa only) were analyzed in the distal femur metaphysis at a threshold of 250. Bone morphometric parameters including bone volume over total volume (BV/TV), trabecular number (Tb.N), trabecular separation (Tb.Sp), trabecular thickness (Tb.Th), and structure model index (SMI) were obtained by analyzing the VOI [21, 22]. The operator conducting the scan analysis was blinded to the treatments associated with the specimen.

#### 2.4.4. Biomechanical Testing

The right femora were tested in three-point bending to evaluate the mechanical properties of the cortical bones at mid-diaphysis using a CSS-4420 material testing machine (Changchun Research Institute for Testing Machines Co. Ltd., China). The femur was placed on the lateral surface on a fixing supporter with two loading points. The distance between the loading points had a fixed length of 20 mm. A preload of 1 N was applied at the medial surface of the diaphysis by lowering a third rounded bar. A constant displacement rate of 1 mm/min was applied until breakage. The force and displacement data were automatically recorded into a computer which was interfaced to the material testing machine and the load-deformation curve was plotted simultaneously. The following mechanical parameters were directly determined from the load-deformation curve: (1) ultimate load (newtons, N), defined as the maximum load, (2) extrinsic stiffness (newtons per millimeter, N/mm), calculated as the slope in the linear region between 40% and 80% of the ultimate load, and (3) energy to ultimate load (millijoules, mJ), defined as the area under the load-deformation curve.

#### 2.4.5. Immunohistochemistry of Femur

Selected femora for immunohistochemical evaluation of OPG and RANKL expression. The selected specimens (*n* = 6/group) were fixed in 10% formalin for 2 days at room temperature and then decalcified in 10% ethylenediaminetetraacetic acid (pH 7.2–7.4, changed every 3 days) for 4 weeks. Decalcified tissues were then washed, dehydrated in gradient alcohol, embedded in paraffin wax, and cut into serial sagittal sections (4 *μ*m thick) through the proximal femur with a microtome (Leica RM2235, Germany). Immunohistochemical localization of OPG and RANKL was carried out using commercially available antibodies according to the manufacturer's suggested protocol (Bioss, Beijing, China). Negative controls were obtained by omitting the primary antibody. Stained sections were examined qualitatively under light microscopy (Leica DM 100, Germany) with Mini See 1.0.9.37 image analyzing system.

### 2.5. Safety Assessment

#### 2.5.1. Serum Analysis

Serum lipid parameters total cholesterol (TC) and triglycerides (TG) levels were determined by using commercial assay kits (Nanjing jiancheng Bioengineering Institute, Nanjing, China). Serum CEA and CA-125 levels were also measured with ELISA kits (R&D Systems Inc.). All of the measurements of ELISA kits were performed according to the protocols provided by the manufacturers.

#### 2.5.2. Histology of Uterus and Mammary Gland

At study end, uteri and mammary glands were fixed in neutral buffered formalin, trimmed, processed, embedded in paraffin, sectioned, and stained with hematoxylin and eosin (H & E) for microscopic examination. Additionally, the interested areas were assayed using a light microscope (Leica DM 100, Germany) equipped with Mini See 1.0.9.37 image analyzing system.

#### 2.5.3. Immunohistochemistry of Uterus and Mammary Gland

Immunostaining was performed using a 5030 kit (MaxVision, Fuzhou, China). Paraffin sections of 4 *μ*m were deparaffinized in toluene and rehydrated through ethanol. A microwave antigen retrieval technique using citrate buffer was used for 10 min. After cooling, the slides were washed with 0.01 mol/L phosphate buffered saline (PBS, pH 7.2–7.4) for three times and 2 min each, and then nonspecific binding sites were blocked by incubation with 10% goat serum for 10 min. Sections were then incubated with a mab-2580 rabbit CEA antibody (MaxVision, Fuzhou, China) diluted 1 : 500 for 1 h at room temperature, washed in PBS buffer for three times and 2 min each, and incubated with biotinylated antimouse secondary antibody for 10 min and thereafter washed in PBS buffer for three times and 2 min each. Diaminobenzidine was used as the chromogen to visualize the biotin/streptavidin-peroxidase complex, under microscope monitoring. Counterstaining was performed using hematoxylin for 1 min. In the negative controls, the primary antibody was omitted. Stained sections were examined qualitatively under light microscopy (Leica DM 100, Germany) with Mini See 1.0.9.37 image analyzing system.

### 2.6. Statistics

All results are presented as mean ± standard deviation (SD). Statistical differences among the sham control, OVX control, and different treatment groups were analyzed using one-way analysis of variance (ANOVA) followed by a post hoc multiple comparison using Fisher's least significant difference (LSD) *t*-test. All calculations were performed using SPSS Version 15.0 for Windows (SPSS, Chicago, IL, USA). In all analyses, a *P* value of <0.05 was considered to be statistically significant.

## 3. Results

### 3.1. Effectiveness Assessment

#### 3.1.1. Serum Biochemical Analysis

Serum ALP levels and bone turnover markers TRACP-5b were assessed at the end of treatment. In Table 1, OVX resulted in a significant increase in serum ALP and TRACP-5b levels compared to sham-operated group. After ECH or E2 administration, serum ALP and TRACP-5b levels were significantly reduced in all three of the ECH groups or E2 group. In addition, the high dose of ECH had the significantly lowest levels of 25.82% and 38.05% as compared with the OVX group (*P* < 0.01). All three doses of ECH treatments had a similar effect as E2 in changing bone turnover markers (*P* < 0.05).

At the end of the protocol, assays for serum OPG and RANKL levels were performed. All doses of ECH treatment groups had significantly higher levels of OPG and OPG/RANKL ratios, as well as a significantly lower RANKL levels than E2-treated or vehicle-treated OVX groups (Table 1). The highest levels of OPG and OPG/RANKL ratios were observed in the ECH-H treatment group, 150.14% and 197.64%, respectively, as compared to OVX group (*P* < 0.01). However, no differences in RANKL levels and the OPG/RANKL ratio were observed in E2 group, as compared with the OVX group.

#### 3.1.2. BMD Assessment

Results of the total femur BMD by DXA were presented in Table 2. As expected, the total femur BMD was decreased by OVX compared with SHAM group (*P* < 0.01). However, All the treated groups significantly increased BMD than OVX group after 12 weeks treatment (*P* < 0.01), but no significant difference was found between treated groups. The three ECH-treated groups increased total femur BMD to 109.25%, 115.27%, and 124.53%, respectively, compared to the OVX group. E2 increased the total femur BMD to 113.82% in comparison to the OVX group.

#### 3.1.3. Microcomputed Tomography (Micro-CT)

**Figure 2:**
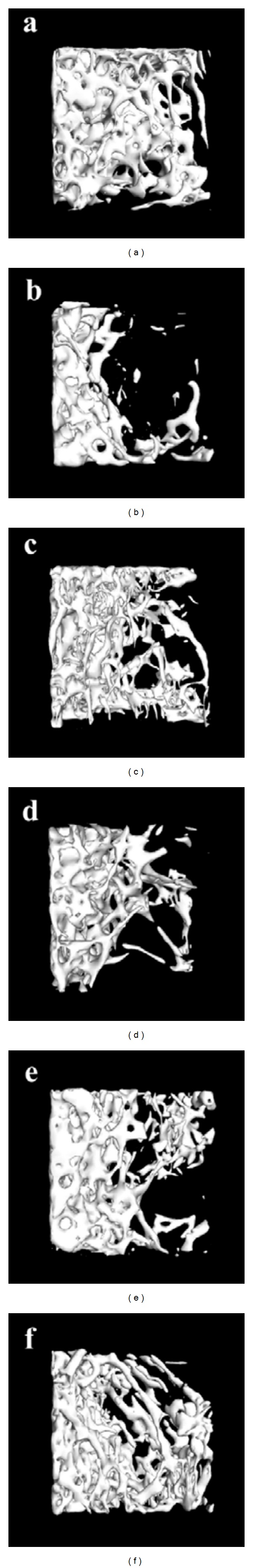
Representative Micro-CT images of trabecular bone microarchitecture in the distal femurs. (a) SHAM group, (b) OVX group, (c) E2 group, (d) ECH-L group, (e) ECH-M group, and (f) ECH-H group. The OVX rats presented notable reduction in the trabecular number, trabecular area compared with the SHAM rats. ECH and E2 partially prevented OVX-induced trabecular bone loss and significantly improved trabecular bone mass and microarchitecture.

Micro-CT scanning is a very accurate method of measuring the vertebral architecture because it quantifies the trabecular structure in three dimensions. The quantitative results of the metaphyseal region close to the growth plate of the distal femur from Micro-CT evaluation were expressed as BV/TV, Tb.N, Tb.Sp, Tb.Th, and SMI in Table 2. Trabecular Micro-CT parameters in Table 2 showed that OVX caused significant decreases in BV/TV, Tb.N, and Tb.Th and increases in Tb.Sp and SMI (*P* < 0.01). Furthermore, the indices BV/TV, Tb.N, and Tb.Th in ECH-H groups were significantly higher than those in OVX group (*P* < 0.01 for BV/TV, and Tb.Th, *P* < 0.05 for Tb.N), and ECH-H treatment significantly increased values of BV/TV by 169.23%, Tb.N by 157.73%, and Tb.Th by 148.23% compared to OVX group. In addition, ECH-H also prevented OVX-induced increase in the levels of Tb.Sp and SMI (*P* < 0.01 for Tb.Sp, *P* < 0.05 for SMI). The preventive effects of ECH on trabecular bone mass and microarchitecture deterioration are further proved by the 3D Micro-CT images (Figures 2(a)–2(f)). OVX group presented notable reduction in the trabecular number and trabecular area when compared with SHAM group. ECH and E2 partially prevented OVX-induced bone loss and significantly improved the trabecular bone mass and microarchitecture.

#### 3.1.4. Three-Point Bending of Femur

The results of biomechanical three-point bending experiment are shown in Table 2. Significant decreases in the ultimate load, stiffness, and energy absorption were observed in OVX group compared with SHAM group (*P* < 0.01). ECH treatment improved bone mechanical strength, evidenced by increased levels of ultimate load, stiffness, and energy absorption (*P* < 0.01 for ultimate load and energy absorption, *P* < 0.05 for stiffness); however, no significant difference was observed among the three ECH-treated groups. The mechanical values of the ECH-H treated group were increased by 55.51% for ultimate load, 34.05% for stiffness, and 183.33% for energy absorption compared to the vehicle treated OVX group (*P* < 0.01). E2 increased ultimate load by 49.51%, stiffness by 28.63%, and energy absorption by 55.56% compared to OVX group, respectively, but its effect on mechanical values were less than the ECH-H group.

### 3.2. Safety Assessment

#### 3.2.1. Serum Analysis

**Figure 3:**
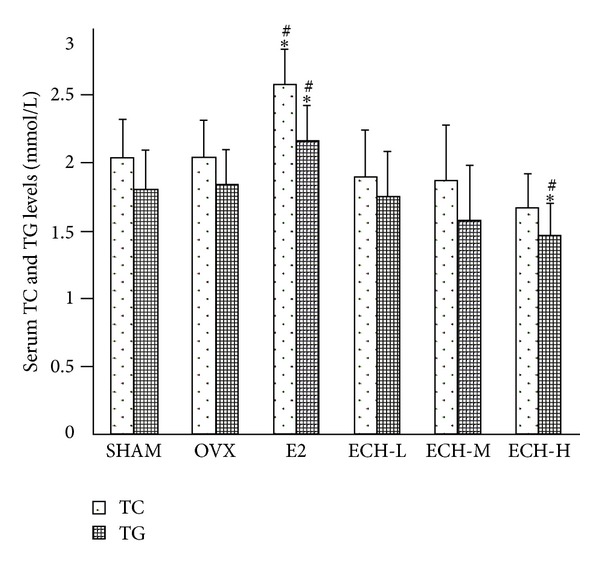
Serum TC and TG levels were determined by assay kit after sacrifice at 12 weeks. Data were expressed as mean ± SD, error bars in the figure are presented as SD, *n* = 8 specimens/group. ^#^ *P* < 0.05 and ^##^ *P* < 0.01 versus sham group, *P < 0.05 and **P < 0.01 versus OVX group at the same time point as evaluated by ANOVA.

Effects on the lipid profile are summarized in Figure 3. At week 12, E2 showed increases from baseline in levels of TC and TG. Three doses of ECH (ECH-L, ECH-M, and ECH-H) reduced TC and TG levels compared to OVX and SHAM groups. In addition, the ECH-H group decreased levels of TG significantly by 30.87% versus OVX (*P* < 0.05), or by 19.07% versus SHAM group (*P* < 0.05). Moreover, the effects with ECH-L and ECH-M on TG levels were marginal.

At the end of the protocol, measurements for serum CEA and CA-125 levels were collected as shown in Table 3. E2 group increased levels of CEA and CA-125 significantly by 54.38% and 59.58% versus OVX (*P* < 0.01), or by 49.53%, 28.87% versus SHAM group (*P* < 0.01). All doses of ECH administration resulted in remarkable reduction in the serum CA-125 levels (*P* < 0.05) compared to SHAM group, but ECH did not show any significant effect on the serum CEA concentrations versus SHAM group or OVX group. In addition, the ECH-H group decreased levels of CA-125 significantly by 29.31% compared to OVX group (*P* < 0.05), or by 42.91% versus SHAM group (*P* < 0.01). All doses of ECH administration resulted in notable reduction in the serum CA-125 and CEA levels (*P* < 0.01) compared to E2 group. Moreover, the ECH-H group decreased levels of CA-125 and CEA significantly by 44.30% and 64.70%, respectively, compared to E2 group.

#### 3.2.2. Histology and Immunohistochemistry of Uterus and Mammary Gland

The most common adverse events of uteri and mammary glands were not observed between all the groups but not the E2 group (Table 3). E2 (50 *μ*g/kg/day) administration for 12 weeks increased endometrium thickness and proliferation of mammary gland as compared to OVX or SHAM group. Furthermore, three mammary glands of CEA expression could also be detected in the E2 group by immunohistochemical analysis, but not uterus. ECH treatment groups were not associated with any abnormity of uterus and mammary gland.

## 4. Discussion

Osteoporosis is a disorder characterized by fragility fractures resulted from loss of bone mass and strength. The remodeling activity is essential to retain bone quality in healthy bone and to produce bones that can adapt appropriately to mechanical stimulus. Because the resorption phases of bone remodeling are short and the period required for osteoblastic replacement of the bone is long, any increase in the rate of bone remodeling will result in a loss of bone mass. Animals develop substantial osteoporosis after ovariectomy within several weeks [17]. The bone loss in ovariectomized (OVX) rat shares many similarities as the process observed in human bodies and therefore serves as a validated and wildly used experimental model of postmenopausal osteoporosis. In this study, we evaluated the effect of ECH on the protection against ovariectomy model of estrogen deficiency induced bone loss in mature rats. E2 was also included as a reference drug for the effect of bone modeling and remodeling. In our study, OVX induced a significant increase of serum TRACP-5b; a surrogate for osteoclast activity was also observed. The decreases in the bone resorption related biomarker [24] agree with previous reports. In line with this, administration of different dose of ECH (30, 90, and 270 mg/kg/day) increased BV/TV, Tb. N. and Tb. Th. and decreased Tb. Sp. and SMI in OVX rats, accompanied by a pronounced upregulation in bone mineral density (BMD) and mechanical properties. Further investigations suggested that ECH administration resulted in lower RANKL level and higher OPG concentration in serum, which led to an enhanced OPG/RANKL ratio. There were no statistically significant differences between ECH groups and SHAM group in lipid parameters (TC and TG) and frequency of endometrial cancer or mammary cancer according to the levels of CEA, CA-125 and histomorphometric analysis of uterus and mammary gland. Altogether, our findings demonstrated the safety and effectiveness of ECH, the mechanism of which may be attributed to the increase of bone formation and the suppression of the bone resorption via upregulating the OPG/RANKL ratio.

Bone maintains its normal structural and functional integrity through continuous remodeling activity, characterized by the equilibrium between osteoblastic bone formation and osteoclastic bone resorption. However, the homeostasis could be disturbed by OVX, resulting in unbalance of bone formation and bone resorption. In a rat ovariectomy model of estrogen deficiency, OVX caused significant increase in bone remodeling after 12 weeks of treatment. This finding agrees with those of other investigators [25, 26]. Furthermore, OVX significantly increased the level of RANKL and decreased the OPG level as well as the OPG/RANKL ratio in serum, demonstrating that the efficiency of ECH in OVX bone loss was mainly associated with the enhancing bone formation and inhibiting bone resorption probably through increasing the ratio of OPG/RANKL.

The trabecular bone microarchitecture is generally considered to be a good predictor of bone mass loss and bone structure deterioration [27]. Micro-CT as a new high resolution digital imaging technique has recently been widely used in the experimental studies to provide detailed quantitative nondestructive analysis of 3D microscopic bone architecture [28]. We evaluated the metaphyseal region close to the growth plate of the distal femur because it is the most recently formed trabecular bone and presumably the most sensitive to dietary factors affecting mineralization. As noted previously in the Micro-CT analysis, normal trabecular bone structure was severely destroyed post-OVX [9]. Consistent with these findings, our results also demonstrated notable trabecular bone deterioration induced by OVX. Further observation suggested that all three doses of ECH treatment had significant effects on trabecular microarchitectural properties, such as BV/TV, Tb.N, Tb.Th, and SMI; however, neither three ECH treatment groups nor E2 group were able to recover the trabecular bone properties completely. These findings are in line with other research articles in which the trabecular structure was unable to be restored after deterioration occurred, suggesting that, in order to prevent trabecular bone loss, the treatment should be initiated at the very first stage after OVX [29].

BMD has been described as a surrogate measure of bone strength and the main contributor to bone quality [30]. In addition, since trabecular bone is more prone to bone loss, in order to evaluate the true effect of treatments on trabecular bones, bone mechanical tests are of great necessity. In this study, we showed a rapid decrease in bone mineral density after OVX as well as more pronounced decrease in the bone strength and we demonstrated that ECH treatment effectively protected against OVX-induced loss of bone mineral density and improved bone biomechanical properties. 

Osteoporosis is mostly caused by increased bone resorption, resulting from increased recruitment, activation, and/or activity of osteoclasts driven by the RANKL signaling [31]. Given the crucial functional roles RANKL and OPG played in regulating physiological and pathological bone turnover. The equilibrium between OPG and RANKL is critical for the homeostasis of bone remodeling. In this study, we observed the estrogen deficiency with a concurrent decrease in the OPG/RANKL ratio, which lead to the increased rate of bone turnover and ultimately accelerated bone loss [32, 33]. Both RANKL and OPG circulate in blood and serum RANKL and OPG measurement has been the subject of numerous studies seeking to relate these levels to various clinical conditions. 

In this study, we observed lower OPG/RANKL ratio caused by increased RANKL levels and reduced plasma OPG in OVX rats. Ovariectomies have been shown to increase RANKL levels in various animal models which lead to the activation of osteoclasts [33]. Our findings in this study are consistent with previous reports that describe decreased serum OPG concentrations in postmenopausal osteoporotic women [34] and the OVX animal model [35] with increased bone turnover TRACP-5b. It has been suggested that elevated OPG in this condition reflects a compensatory reaction. These results also agreed with our *in vitro* data showing that ECH significantly increases OPG levels and inhibits RANKL expression in osteoblasts [16]. Overall, these results seemed to indicate that ECH is exerting its effect through suppressing the activity of osteoclasts and enhancing the function of osteoblasts via increasing the ratio of OPG/RANKL and therefore attenuating osteoporosis in OVX rat models.

Cancer Antigen 125 (CA-125) is a tumor marker or biomarker that may be elevated in the blood of some specific types of cancers, elevated marker not only for ovarian cancer in serum but also in other malignant cancers, including those originating in the endometrium, fallopian tubes, lungs, breast, and gastrointestinal tract; CEA, one of the first oncofetal antigens to be described and exploited clinically, is associated with the plasma membrane of tumor cells, from which it may be released into the blood, and elevated CEA levels were not only identified in colon cancer but also found in a variety of cancers including pancreatic, gastric, lung, and breast ones [36]. Moreover, Ahmadi et al. determined the effects of *Salvia officinalis* extract on serum level of CEA in male rats and showed that appropriate dose of *Salvia officinalis* extract can decrease serum level of CEA, on which medicinal application of this extract particularly in cancers accompanied by CEA increased serum level is conceivable [37]. Zhou et al. explored the effects of the extracts of *Salvia miltiorrhiza* Bunge (SMB) on the serum levels of CA-125 in the peritoneal fluids of rat endometriosis models and found that SMB decreased the serum CA-125 levels [38]. Therefore, we tested the serum CEA, CA-125 levels by using ELISA kits to testify the potential effect of E2 and ECH on mammary glands and uteri in OVX rats.

As implicated through our results, treatment of ECH can also significantly diminish the markers of cancer and cardiovascular disorders. These promising findings highlighted the great advantages of ECH in avoiding undesirable side effects in postmenopausal osteoporotic patients. Compared to the E2 group (50 *μ*g/kg/day, six times a week), which increased TC, TG levels and expression of tumor markers, the ECH treatment groups displays improved lipid parameters and reduced tumor markers CA-125 and CEA expression. Interestingly, there were many manuscripts reported that E2 (20~200 *μ*g/kg/day) lowers rat TC and TG by oral administration or injection for a period of 4~5 weeks, but in our study, E2 group increased TC and TG levels significantly after 12-week administration which agreed with Lee et al. [39]. We implied that the higher dosage, longer, and frequent treatment may account for the discrepancy with other previous researchers, but the actual reason for this difference will be studied in the future. 

## 5. Conclusions

Our study is the first to report that ECH administration could safely and effectively prevent OVX-induced bone loss through increasing OPG/RANKL ration, which was evidenced by the serum biochemical analysis, bone mineral density assay, Micro-CT analysis, biomechanical properties test, immunohistochemical evaluation, and histology assessment results. These findings shed some light on the potential of ECH, as a natural derived compound, to be developed into a safe and effective agent for prevention or treatment of osteoporosis in postmenopausal osteoporotic women.

## Figures and Tables

**Table 1:** Serum parameters after 12-week administration of ECH.

Group	SHAM	OVX	E2	ECH-L	ECH-M	ECH-H
ALP (U/100 mL)	10.96 ± 1.36	14.76 ± 2.86^##^	11.66 ± 1.88*	12.20 ± 1.22*	12.00 ± 1.56*	10.95 ± 1.97**
TRACP-5b (U/L)	0.306 ± 0.026	0.367 ± 0.054^#^	0.294 ± 0.054*	0.277 ± 0.084*	0.231 ± 0.088**	0.227 ± 0.082**
OPG (pg/mL)	610.32 ± 85.29	516.36 ± 65.35^#^	621.41 ± 121.78*	631.77 ± 131.64*	729.42 ± 122.59**	775.29 ± 179.87**
RANKL (pg/mL)	6.44 ± 0.55	7.38 ± 0.83^#^	7.19 ± 1.75	6.38 ± 0.58*	6.15 ± 1.01*	5.75 ± 1.21**
OPG/RANKL ratio	95.09 ± 13.44	70.32 ± 8.53^##^	92.74 ± 35.72	100.75 ± 28.50*	121.41 ± 29.23**	138.98 ± 39.16**

The data are expressed as mean ± SD, *n* = 8. ^#^ *P* < 0.05 and ^##^ *P* < 0.01 versus SHAM group, *P < 0.05 and **P < 0.01 versus OVX group at the same time point as evaluated by ANOVA.

**Table 2:** BMD, Micro-CT properties of femoral trabeculae and biomechanical test of femur after 12-week administration of ECH.

Group	SHAM	OVX	E2	ECH-L	ECH-M	ECH-H
BMD (g/cm^2^)	0.28 ± 0.01	0.22 ± 0.01^##^	0.26 ± 0.01**	0.25 ± 0.01**	0.26 ± 0.01**	0.28 ± 0.01**
BV/TV (%)	58.71 ± 6.38	16.42 ± 3.44^##^	34.52 ± 3.38**	23.22 ± 4.07*	26.12 ± 2.02**	28.80 ± 2.53**
Tb.N (1/mm)	3.88 ± 0.25	2.18 ± 0.48^##^	3.01 ± 0.10*	2.76 ± 0.58	3.02 ± 0.38*	3.12 ± 0.41*
Tb.Sp (*μ*m)	106.52 ± 14.78	452.02 ± 65.48^##^	219.83 ± 9.99**	355.04 ± 41.53*	321.98 ± 28.59*	279.57 ± 23.15**
Tb.Th (*μ*m)	152.28 ± 22.30	84.05 ± 7.19^##^	114.24 ± 8.13**	97.12 ± 7.47*	112.06 ± 20.16*	115.32 ± 10.79**
SMI	0.78 ± 0.12	2.77 ± 0.49^##^	1.41 ± 0.25**	2.11 ± 0.15*	1.90 ± 0.43*	1.86 ± 0.17*
Maximum load (N)	87.54 ± 5.86	60.00 ± 3.35^##^	89.71 ± 6.45**	83.05 ± 7.81**	83.70 ± 6.49**	93.31 ± 13.66**
Stiffness (N/mm)	190.98 ± 36.03	147.56 ± 12.54^#^	189.81 ± 18.33**	173.17 ± 24.52*	178.67 ± 18,55**	197.79 ± 32.05**
Energy to ultimate load (millijoules, mJ)	17.33 ± 2.58	12.00 ± 1.79^##^	18.67 ± 3.01**	25.67 ± 2.88**	25.83 ± 3.13**	34.00 ± 4.65**

The data are expressed as mean ± SD, *n* = 6. ^#^ *P* < 0.05 and ^##^ *P* < 0.01 versus SHAM group, *P < 0.05 and **P < 0.01 versus OVX group at the same time point as evaluated by ANOVA.

**Table 3:** Serum CEA, CA-125 levels and incidence of principal microscopic pharmacologic effects of ECH and E2 on female rat's uteri and mammary glands.

Group	SHAM	OVX	E2	ECH-L	ECH-M	ECH-H
Serum parameters	*n* = 8/group					
Serum CEA (pg/mL)	341.82 ± 32.94	331.08 ± 36.97	511.11 ± 62.39^##∗∗^	346.03 ± 28.62	342.22 ± 42.57	330.67 ± 60.93
Serum CA-125 (U/mL)	1.89 ± 0.40	1.52 ± 0.39	2.43 ± 0.24^##∗∗^	1.37 ± 0.29*	1.32 ± 0.41*	1.08 ± 0.27^#∗∗^
Uteri	*n* = 6/group					
Endometrium thickness changes	++	+	+++	+	+	+
Immunohistochemical analysis of CEA	—	—	—	—	—	—
Mammary glands	*n* = 6/group					
Proliferative changes	+	—	++	—	—	—
Tubular changes	—	—	—	—	—	—
Ductal intraepithelial neoplasia	—	—	—	—	—	—
Carcinoma of breast	—	—	—	—	—	—
Immunohistochemical analysis of CEA	—	—	+	—	—	—

The data are expressed as mean ± SD, *n* = 8. ^#^ *P* < 0.05 and ^##^ *P* < 0.01 versus SHAM group, *P < 0.05 and **P < 0.01 versus OVX group at the same time point as evaluated by ANOVA.

+: minimal; ++: mild; +++: marked; —: not observed, the qualitative system according to [23] used previously.

## Abbreviations

ALP: Alkaline phosphatase
BMD: Bone mineral density
BV/TV: Bone volume fraction
CA-125: Cancer antigen 125
CEA: Carcinoembryonic antigen
CMC-Na: Carboxymethylcellulose sodium
E2: 17*β*-estradiol
ECH: Echinacoside
ELISA: Enzyme-linked immunosorbent assay
ERT: Estrogen replacement therapy
i.g.: Intragastric
i.p.: Intraperitoneal
Micro-CT: Microcomputed tomography
OPG: Osteoprotegerin
OVX: Ovariectomized
PBS: Phosphate buffered saline
RANK: Receptor activator of nuclear factor-*κ*B
RANKL: Receptor activator of nuclear factor-*κ*B ligand
SMI: Structure model index
Tb.N: Trabeculae number
Tb.Sp: Trabeculae separation
Tb.Th: Trabeculae thickness
TC: Total cholesterol
TG: Triglycerides
TRACP-5b: Tartrate-resistant acid phosphatase 5b.
